# A cost-effective machine learning-based method for preeclampsia risk assessment and driver genes discovery

**DOI:** 10.1186/s13578-023-00991-y

**Published:** 2023-02-28

**Authors:** Hao Wang, Zhaoyue Zhang, Haicheng Li, Jinzhao Li, Hanshuang Li, Mingzhu Liu, Pengfei Liang, Qilemuge Xi, Yongqiang Xing, Lei Yang, Yongchun Zuo

**Affiliations:** 1 The State Key Laboratory of Reproductive Regulation and Breeding of Grassland Livestock, College of Life Sciences, Inner Mongolia University, Hohhot, 010070 China; 2 Digital College, Inner Mongolia Intelligent Union Big Data Academy, Inner Mongolia Wesure Date Technology Co., Ltd., Hohhot, 010010 China; 3 School of Life Science and Technology, Center for Informational Biology, University of Electronic Science and Technology of China, Chengdu, 610054 China; 4 School of Life Science and Technology, Inner Mongolia University of Science and Technology, Baotou, 014010 China; 5 College of Bioinformatics Science and Technology, Harbin Medical University, Harbin, 150081 China

**Keywords:** Preeclampsia risk, Machine learning, Feature selection, Marker genes, Web server

## Abstract

**Background:**

The placenta, as a unique exchange organ between mother and fetus, is essential for successful human pregnancy and fetal health. Preeclampsia (PE) caused by placental dysfunction contributes to both maternal and infant morbidity and mortality. Accurate identification of PE patients plays a vital role in the formulation of treatment plans. However, the traditional clinical methods of PE have a high misdiagnosis rate.

**Results:**

Here, we first designed a computational biology method that used single-cell transcriptome (scRNA-seq) of healthy pregnancy (38 wk) and early-onset PE (28–32 wk) to identify pathological cell subpopulations and predict PE risk. Based on machine learning methods and feature selection techniques, we observed that the Tuning ReliefF (TURF) score hybrid with XGBoost (TURF_XGB) achieved optimal performance, with 92.61% accuracy and 92.46% recall for classifying nine cell subpopulations of healthy placentas. Biological landscapes of placenta heterogeneity could be mapped by the 110 marker genes screened by TURF_XGB, which revealed the superiority of the TURF feature mining. Moreover, we processed the PE dataset with LASSO to obtain 497 biomarkers. Integration analysis of the above two gene sets revealed that dendritic cells were closely associated with early-onset PE, and *C1QB* and *C1QC* might drive preeclampsia by mediating inflammation. In addition, an ensemble model-based risk stratification card was developed to classify preeclampsia patients, and its area under the receiver operating characteristic curve (AUC) could reach 0.99. For broader accessibility, we designed an accessible online web server (http://bioinfor.imu.edu.cn/placenta).

**Conclusion:**

Single-cell transcriptome-based preeclampsia risk assessment using an ensemble machine learning framework is a valuable asset for clinical decision-making. *C1QB* and *C1QC* may be involved in the development and progression of early-onset PE by affecting the complement and coagulation cascades pathway that mediate inflammation, which has important implications for better understanding the pathogenesis of PE.

**Supplementary Information:**

The online version contains supplementary material available at 10.1186/s13578-023-00991-y.

## Background

The placenta plays a central role in the health of the fetus and mother, profoundly affecting humankind’s future well-being [1]. The dysregulation of the placenta may lead to adverse pregnancy, such as preeclampsia, the birth of small gestational age neonates, fetal growth restriction and intrauterine placental abruption, which significantly influence the lifelong health of mothers and offspring [2, 3]. Preeclampsia is one of the most terrifying complications of pregnancy that has severe morbidity and mortality [4]. The statistics show that preeclampsia affects an estimated 4–5% of pregnancies and leads to more than 70,000 maternal deaths and 500,000 fetal deaths annually [5].

The current diagnosis of preeclampsia is based on the examination of hypertension (> 140/90 mm Hg) and proteinuria (> 0.3 g/24 h) after 20 weeks of gestation [6]. These clinical indicators, however, have a high misdiagnosis rate for preeclampsia, which can add to medical expenses and lead to patient anxiety. In recent years, animal models have demonstrated that placental dysfunction including oxidative stress, abnormal natural killer cells at the maternal-fetal interface, and genetic factors, is strongly associated with preeclampsia [4]. Tsang and colleagues found the cellular dysfunction of extravillous trophoblasts in preeclampsia placentas, suggesting a potential association between disorders of placental cell subpopulations and preeclampsia [7]. The analysis of the interwoven relationship between placental cell subpopulations and preeclampsia will be helpful for the diagnosis of preeclampsia.

High-throughput sequencing technology is a powerful tool for revealing cellular heterogeneity and has been employed to reveal the placenta’s cellular composition [8] and predict pregnancy complications [9]. Liu et al. performed single-cell transcriptome (scRNA-seq) of human placentas from the first and second-trimester and identified new subtypes of trophoblasts, Hofbauer cells, and mesenchymal stromal cells [10]. Besides, changes in gene expression associated with the pathogenesis of preeclampsia are readily detected throughout pregnancy. Moufarrej et al. found changes in cell-free RNA (cfRNA) expression between normal and preeclamptic mothers [11]. Based on comprehensive transcriptome data, Rasmussen et al. further demonstrated the ability of plasma cfRNA to reveal patterns of normal pregnancy progression and determine the risk of developing preeclampsia months before the clinical presentation [12]. They constructed a machine learning model to predict preeclampsia with a sensitivity of 75%. Ngo et al. found that the measurement of nine cfRNA transcripts in maternal blood can predict gestational age with comparable accuracy to ultrasound but at a substantially lower cost [13]. The inherent complexity and scale of omics data have encouraged researchers to build automated analytical models and solve associated tasks by machine learning [14–21]. Nevertheless, to our knowledge, the study of identifying placental cell subpopulations and assessing the risk of PE based on scRNA-seq expression profiles implemented by machine learning is still poor.

**Fig. 1:**
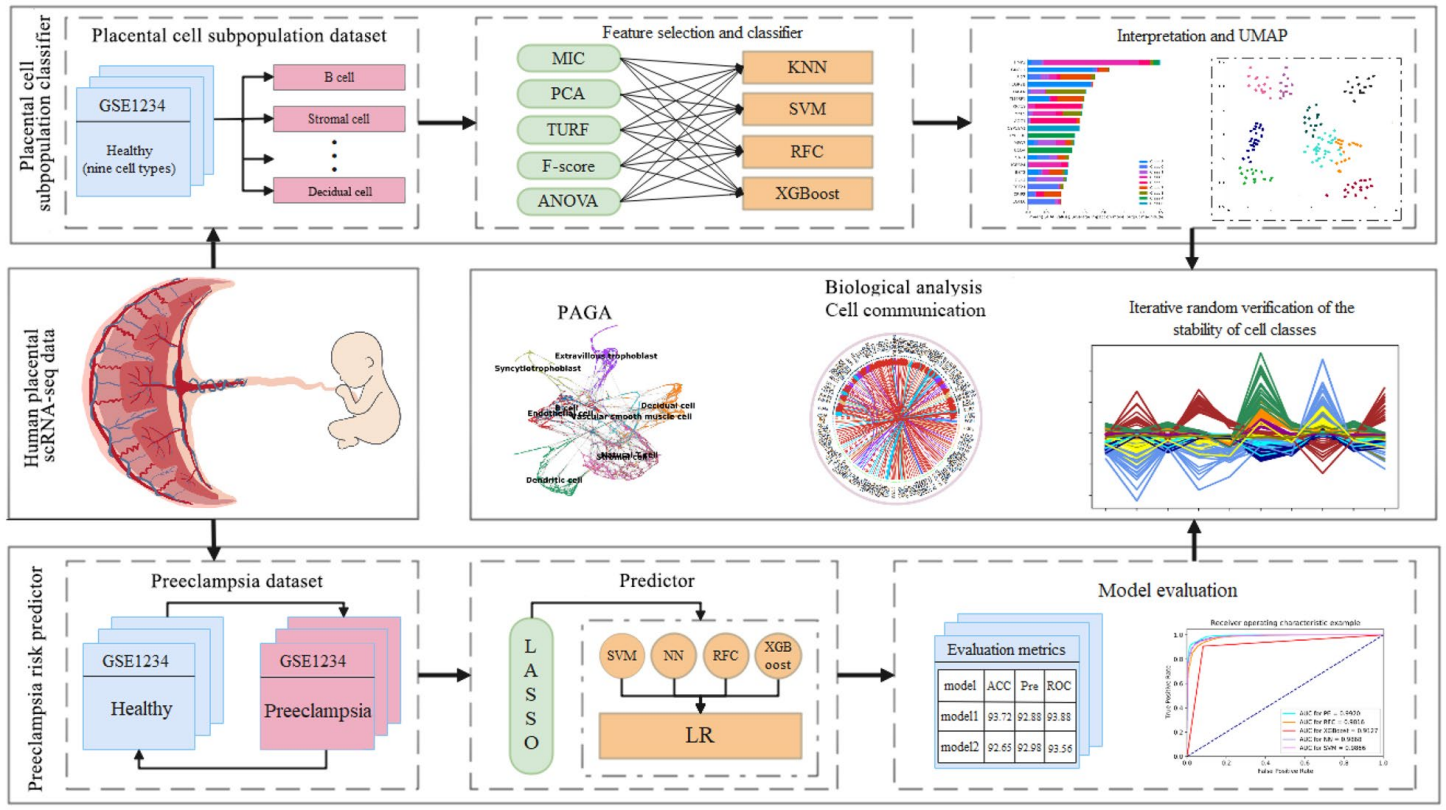
The workflow of construction and validation for the computational framework

In this study, machine learning algorithms were employed to identify preeclampsia biomarkers and assess the risk of preeclampsia based on scRNA-seq data (Fig. 1 and Additional file 1: Fig. S1). A series of prediction analyses demonstrated that the Tuning ReliefF (TURF) score combined with the eXtreme Gradient Boosting (XGBoost) strategy achieves better classification performance on the cell identification, and that the identity of nine cell subpopulations in the placenta could be described using only 110 marker genes. Moreover, we found some new biomarkers that might help biologists better understand placental cell subpopulations and pathological differences between early-onset PE patients and healthy controls. We developed an ensemble model-based risk stratification card to classify early-onset PE patients. By employing this card for PE patients, immediate intervention and treatment can be implemented at the optimum time, and the overall mortality of patients can be significantly reduced. Based on the proposed model, the webserver for predicting placental cell subpopulations and evaluating the risk of PE was established and was freely accessible at http://bioinfor.imu.edu.cn/placenta.

## Results

### Identify marker genes of placental cell subpopulations by machine learning

**Fig. 2:**
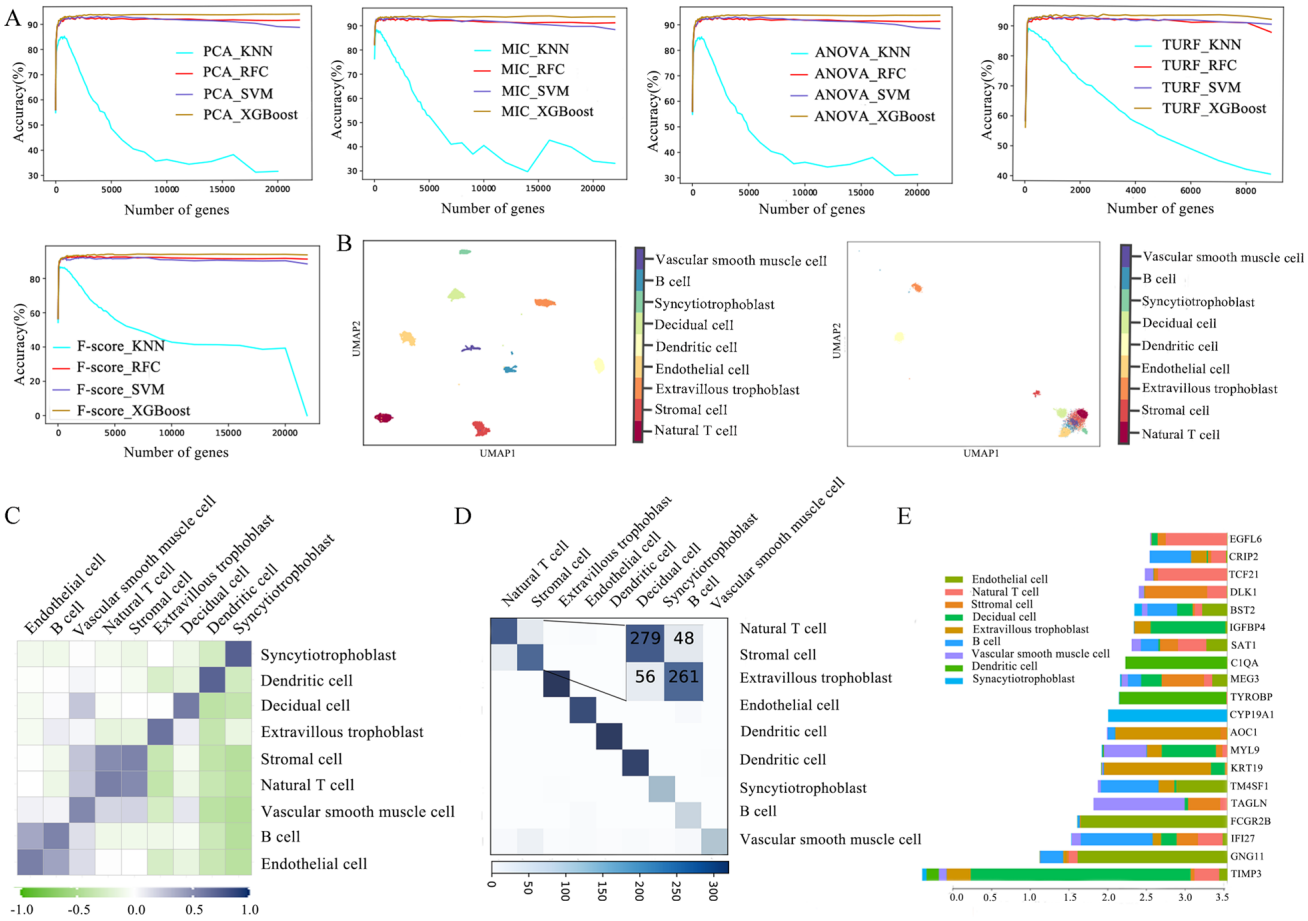
Evaluation and analysis of machine learning classifiers based on different feature selection strategies. **A** IFS results of five feature selection strategies in four machine learning algorithms. **B** UMAP shows the clustering of nine placental cell subpopulations in all gene sets (right) and TURF optimal gene sets (left). **C** The heatmap shows the correlation of subpopulations of placental cells. **D** Based on the TURF optimal gene set, the Confusion matrix of XGBoost on the independent dataset. **E** The bar graph shows the mean absolute value of the SHAP values of the first 20 genes for the TURF_XGB

For identifying marker genes related to nine placental cell subpopulations, five feature selection methods (maximal information coefficient: MIC, principal component analysis: PCA, F-score, tuning relief: TURF, analysis of variance: ANOVA) were employed to evaluate the importance of the 35,636 genes, and genes with importance score less than or equal to zero were excluded. The MIC, ANOVA, PCA, and F-score extracted 21,981 important genes, while the TURF identified 8878 important genes. Next, the machine learning models combined with incremental feature selection (IFS) were used to determine the optimal gene subsets and the best machine learning model. The single-cell gene expression profiles of important genes were used as input features to train four machine learning models (Support Vector Machine: SVM, Random Forest Classifier: RFC, XGBoost, K-Nearest Neighbor: KNN) with five-fold cross-validation (Fig. 2A and Additional file 1: Table S1).

The results of the independent test set showed that TURF combined with XGBoost (TURF_XGB) with the top 110 genes achieved optimal performance with accuracy, precision, recall and F1-measure of 92.61%, 92.98%, 92.46 and 92.65%, which could be used to identify placental cell populations (Table 1). Notably, KNN’s performance on placental cell subpopulation classification was significantly poorer than the other three machine learning models. This can be attributed to KNN is not good at handling single-cell datasets with high feature dimensions single-cell datasets [22, 23].

**Table 1:** Performance of five feature selection strategies for identifying placental cell subpopulations on four machine learning models (Independent dataset)

Base classifier	Feature selection	Feature numbers	Accuracy (%)	Precision (%)	Recall (%)	F1 measure (%)
KNN	PCA	160	71.67	79.69	68.57	71.32
RFC	PCA	3000	91.13	93.43	90.80	91.87
SVM	PCA	1200	90.12	91.93	89.98	90.87
XGBoost	PCA	2000	92.11	93.03	91.72	92.32
KNN	MIC	210	88.39	90.43	88.40	88.83
RFC	MIC	260	92.40	93.63	92.28	92.83
SVM	MIC	160	92.65	93.22	92.89	93.14
XGBoost	MIC	310	93.07	93.76	92.87	93.25
KNN	TURF	110	87.88	90.61	86.80	87.85
RFC	TURF	310	92.35	93.90	92.24	92.86
SVM	TURF	210	92.31	93.23	92.48	92.75
XGBoost	TURF	110	92.61	92.98	92.46	92.65
KNN	F-score	310	85.43	88.74	85.71	85.97
RFC	F-score	610	92.02	93.43	92.03	92.62
SVM	F-score	710	92.02	92.87	92.37	92.54
XGBoost	F-score	410	92.10	93.04	92.31	92.64
KNN	ANOVA	360	84.00	87.28	83.99	83.91
RFC	ANOVA	810	92.10	93.75	91.95	92.65
SVM	ANOVA	710	92.02	93.33	92.51	92.83
XGBoost	ANOVA	460	92.11	93.24	92.22	92.67

Furthermore, the Uniform Manifold Approximation and Projection (UMAP) [24] and correlation analysis showed that the overall performance of the 110 marker genes is significantly better than all genes (Fig. 2B, C). We successfully captured some reported population-specific marker genes, such as *CGA, COL1A1*, *FAR2*, and *CYP19A* [25, 26]. In addition, several novel marker genes were identified, such as *IDO1*, *STMN1*, *CRIP2*, *COX7A1*, and *CCNDBP1* (Additional file 1: Fig. S2). These genes can be used to classify placental cell subpopulations and provide some guidelines for further biological findings.

The confusion matrix further validated the predictive performance of the model for each cell subpopulation, and the low misclassification rate demonstrated the power of the XGBoost model (Fig. 2D). How individual genes influence XGBoost to make decisions was determined by calculating the average absolute Shapley’s addition operation (SHAP) values for 110 genes. For example, the genes *C1QA* and *CYP19A1* have strong positive effects on model prediction of decidual cells and syncytiotrophoblast (Fig. 2E).

### Biological interpretation of marker genes for cell subpopulations

**Fig. 3:**
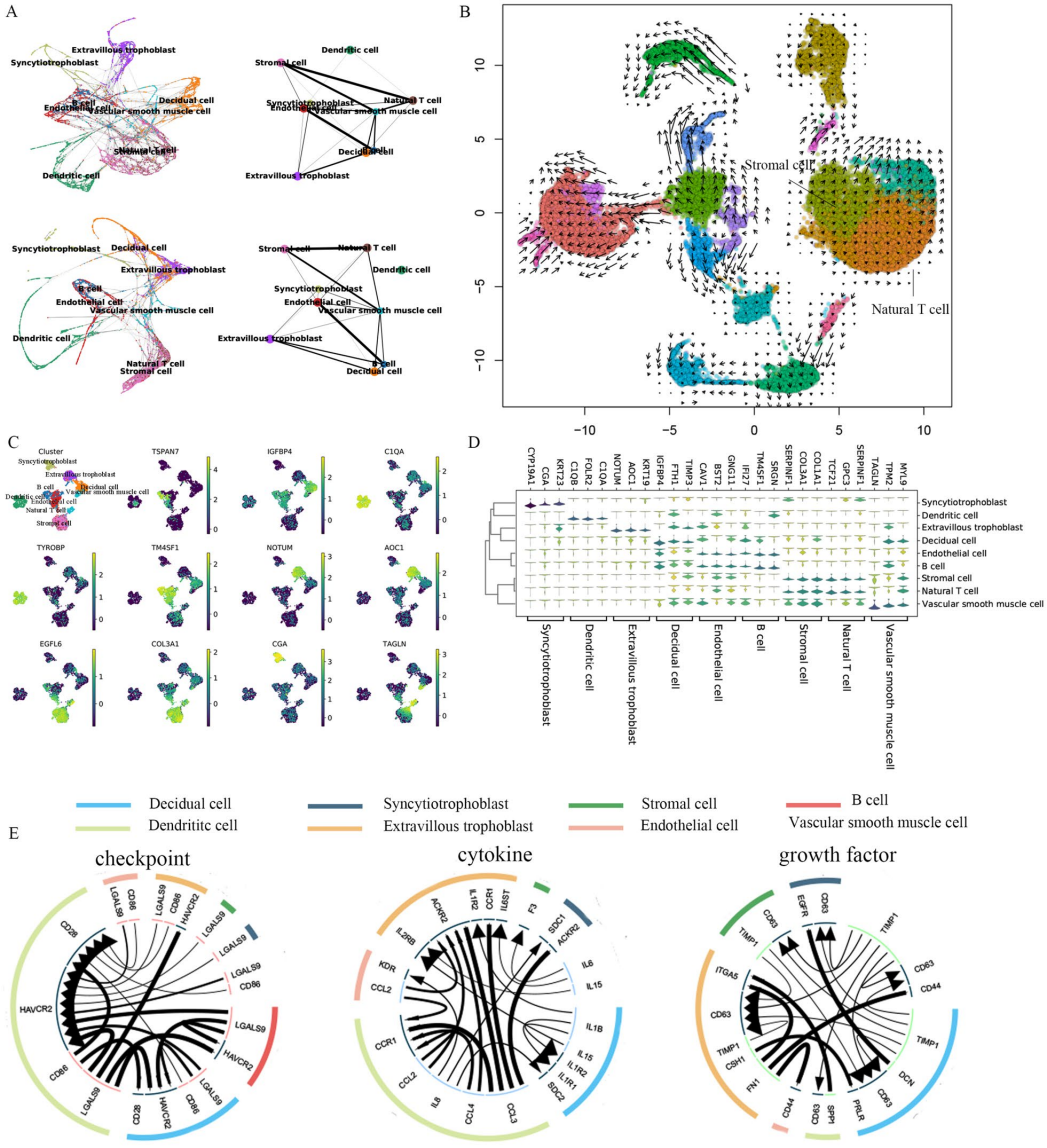
Biological analysis of TURF optimal gene set. **A** Gene expression trajectory analysis of nine placental cell subpopulations using PAGA, color by cell lineages (up: all genes, down: TURF optimal gene set). **B** Velocities derived from the dynamical model for placenta subpopulations are visualized as streamlines in a UMAP-based embedding. **C** Expression patterns of marker genes in different subpopulations of placental cells in the TURF optimal gene set. **D** High expression marker genes screened by Scanpy. **E** Circles plot showing highly expressed ligand-receptor interactions in the TURF optimal gene set

Further, we explored the representational capacity of 110 genes in the biological landscape. Partition-based graphical abstraction (PAGA) was applied to 110 genes and all genes to describe the relationships among cell subpopulations [27]. Interestingly, the same topological structure was shown, such as the strong connections between natural T and stromal cells, which further demonstrated that TURF_XGB captured marker genes and removed noise information (Fig. 3A). By embedding the RNA velocities [28] of all samples in UMAP, we revealed the complex dynamics among placental cell subpopulations. We found that natural T and stromal cells mainly existed in a stable state and had similar migration trajectories, consistent with the PAGA results (Fig. 3B).

Then, the expression levels of 110 genes were quantified using Seurat to identify expressed features of marker genes for subpopulations of placental cells. For example, *IDO1* is specifically expressed in endothelial cells [29], *CGA* is strongly expressed in syncytiotrophoblast cells and *ACAT2* is associated with vascular smooth muscle cells [30] (Fig. 3C and Additional file 1: Fig. S2). Using multiple genes to characterize placental cell subpopulations allowed for greater ability to mark. Based on 110 genes screened by TURF_XGB, the top three specific genes with the highest expression levels in each cell subpopulation were selected using Scanpy (Fig. 3D). Further, we compared the expression levels of the top 12 genes in each cell subpopulation with the overall expression levels of these genes in the remaining eight cell clusters (Additional file 1: Fig. S3). In summary, we presented potential biomarkers for nine placental cell subpopulations.

Crosstalk between placental cell subpopulations may play a critical role in placental development, metastasis, and therapy. Based on the 110 genes obtained above, we used iTALK to analyze and visualize ligand receptor-mediated intercellular crosstalk signaling in nine subpopulations of placental cells [31]. Network analysis showed ligand-receptor pairings between highly transcribed genes on decidual and vascular smooth muscle cells and the most significantly expressed genes on dendritic cells and extravillous trophoblasts (Fig. 3E). Overall, dendritic cells were major ligands that played an essential role in regulating other lineages. However, the natural T cell communication was not captured.

Furthermore, enrichment analysis of 110 genes indicated that *EZR*, *HMGB3*, *TMEM176B*, *COL3A1*, and *C1QC* genes were the main contributors to the negative regulation involved in immune system processes (Additional file 1: Fig. S4). Kyoto Encyclopedia of Genes and Genomes (KEGG) pathway analysis showed that these genes are mainly enriched in the pathways of “complement and coagulation cascades,“ “ferroptosis,“ “mineral absorption,“ “proteoglycans in cancer” and “staphylococcus aureus infection” (Additional file 1: Fig. S4).

### Identify discriminating genes of preeclampsia by LASSO regression

**Fig. 4:**
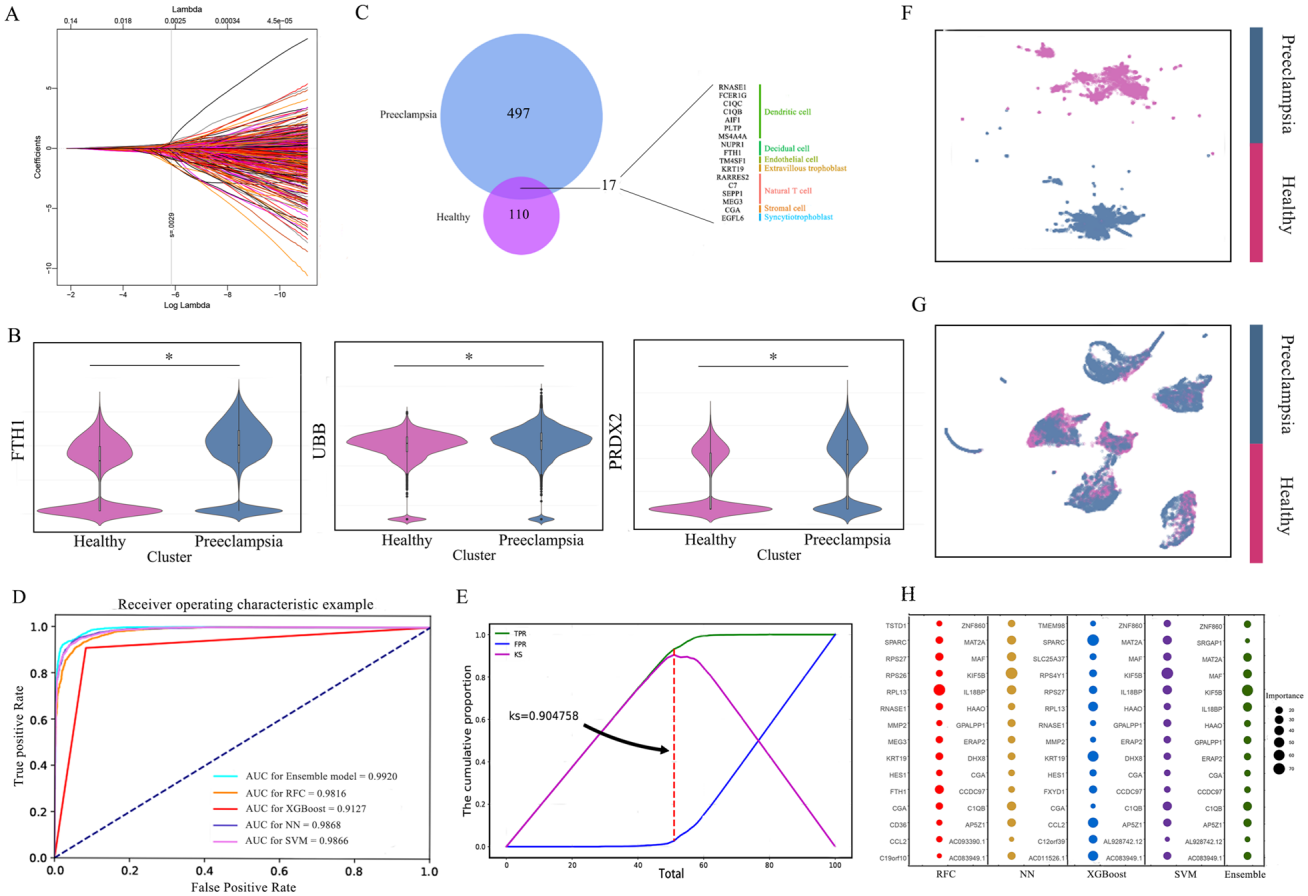
Performance and gene analysis of the model in predicting healthy population and preeclampsia patients. **A** LASSO for gene selection. The vertical dotted line shows the best lambda value of 0.0029 selected through fivefold cross-validation. **B** Differentially expressed genes between preeclampsia placenta and normal placenta. *P-value < 0.001, t-test. **C** Overlapping genes between molecular markers of placenta subpopulations and preeclampsia pathology. **D** ROC curve of preeclampsia risk assessment model. **E** KS curve for preeclampsia risk score card. **F**, **G** Based on the ensemble model, the clustering effect of the LASSO optimal gene set and all genes is compared (**F** is the optimal LASSO optimal genes, **G** is all genes). **H** The importance of genes identified by different preeclampsia risk models, and the size of the circle represents the value of relative importance

Cellular abnormalities in the placenta of PE affect cell renewal, and the origin of the abnormal cells can be uncovered by comparing the expression levels of specific genes in placental cell subpopulations of PE patients with those of healthy pregnant controls [7]. To reveal abnormal cell subpopulations, we constructed a sufficiently large PE dataset (Additional file 1: Table S4). Based on these single-cell expression profiles, 497 potential PE marker genes were identified by LASSO regression (Fig. 4A). Some of these genes have been discussed to have potential as PE marker genes, including *UBB*, *RARRES2*, *PRDX2*, *C19orf10*, *KRT19*, *RPL13*, *FTH1*, *DPM2*, and *DHX29* (Fig. 4B) [32]. A total of 17 in 497 genes from the LASSO screen overlapped with 110 maker genes screened by the TURF_XGB, indicating that the expression levels of these 17 genes were abnormal between normal pregnancy and PE. Seven of the 17 genes were highly expressed in dendritic cells, indicating that dendritic cells were closely associated with early-onset PE and were potential pathogenic cells (Fig. 4C).

Furthermore, KEGG pathway enrichment analysis of 497 genes revealed that they were mainly involved in complement and coagulation cascade and ECM-receptor interactions (Additional file 1: Fig. S5). It was worth noting that *C1QB*, *C1QC*, and *C7* of the 17 genes obtained above were associated with complement and coagulation cascades. Complement and coagulation cascades activation is the main pathophysiological pathway in PE revealed by related studies [33–35]. Thus, *C1QB*, *C1QC*, and *C7* may participate in the occurrence and development of early-onset PE by affecting the complement and coagulation cascades pathway that mediate inflammation, similar to recent findings [36].

### The risk stratification card of preeclampsia based on an ensemble model

As gene expression changes associated with preeclampsia pathogenesis across gestation were readily detected [11], we sought to build a risk prediction model to assist physicians in diagnosing mothers at risk for early-onset PE. We employed four machine learning models (multilayer perceptron: MLP, SVM, RFC, and XGBoost) to predict early-onset PE based on 497 genes screened by LASSO (Table 2). To further improve the performance of the risk prediction model, we integrated an ensemble model of four basic classifiers (SVM, MLP, RFC, and XGBoost) and then fitted logistic regression [37–39]. The performance metrics for the four machine learning models and the proposed ensemble model are presented in Table 2, and the AUC is shown in Fig. 4D. We observed that the ensemble model outperformed the other machine learning models with 94.62% accuracy and 0.99 AUC (Table 2; Fig. 4D). To improve the convenience and flexibility of the model in clinical application, we used the Kolmogorov-Smirnov (KS) curve to determine the suitable threshold for the risk stratification card for the patient. We set equal frequency bins based on sample size into five risk stratification corresponding to very high, high, normal, low, and very low-risk levels (Fig. 4E and Additional file 1: Table S2). Besides, the clustering effect of the LASSO gene set was significantly improved compared with all genes (Fig. 4F and G), which further verified the representation capability of LASSO’s feature selection strategy.

**Table 2:** Performance of machine learning models in identifying patients with preeclampsia (Independent dataset)

Base classifier	Feature selection	Feature number	Accuracy (%)	Precision (%)	Recall (%)	F1-measure (%)
MLP	LASSO	497	94.30	94.95	94.35	94.65
SVM	LASSO	497	94.28	95.33	93.36	94.58
RFC	LASSO	497	92.76	93.62	92.76	93.18
XGBoost	LASSO	497	91.23	92.71	90.71	91.70
Ensemble model	LASSO	497	94.62	95.83	94.56	94.95

Further, we used the SHAP framework to determine which genes had the greatest impact on the model predictions. The SHAP scores displayed the contribution of the top 15 feature values for decreasing or increasing the prediction value assigned to each cell. Among them, *CGA*, *MAF*, *C1QB*, *KIF5B*, *HAAO*, *AP5Z1*, and *1L1BP* showed excellent discrimination ability in multiple models (Fig. 4H). Notably, the gene *C1QB* was also identified in the models, highlighting the imbalance of *C1QB* between healthy pregnancies and preeclampsia.

## Webserver

Based on our proposed machine learning models of placental cell subpopulations and early-onset preeclampsia, an online predictor called iPlacenta was established to classify placental cell populations and assess the risk of preeclampsia. A step-by-step guide is given below.

**Fig. 5:**
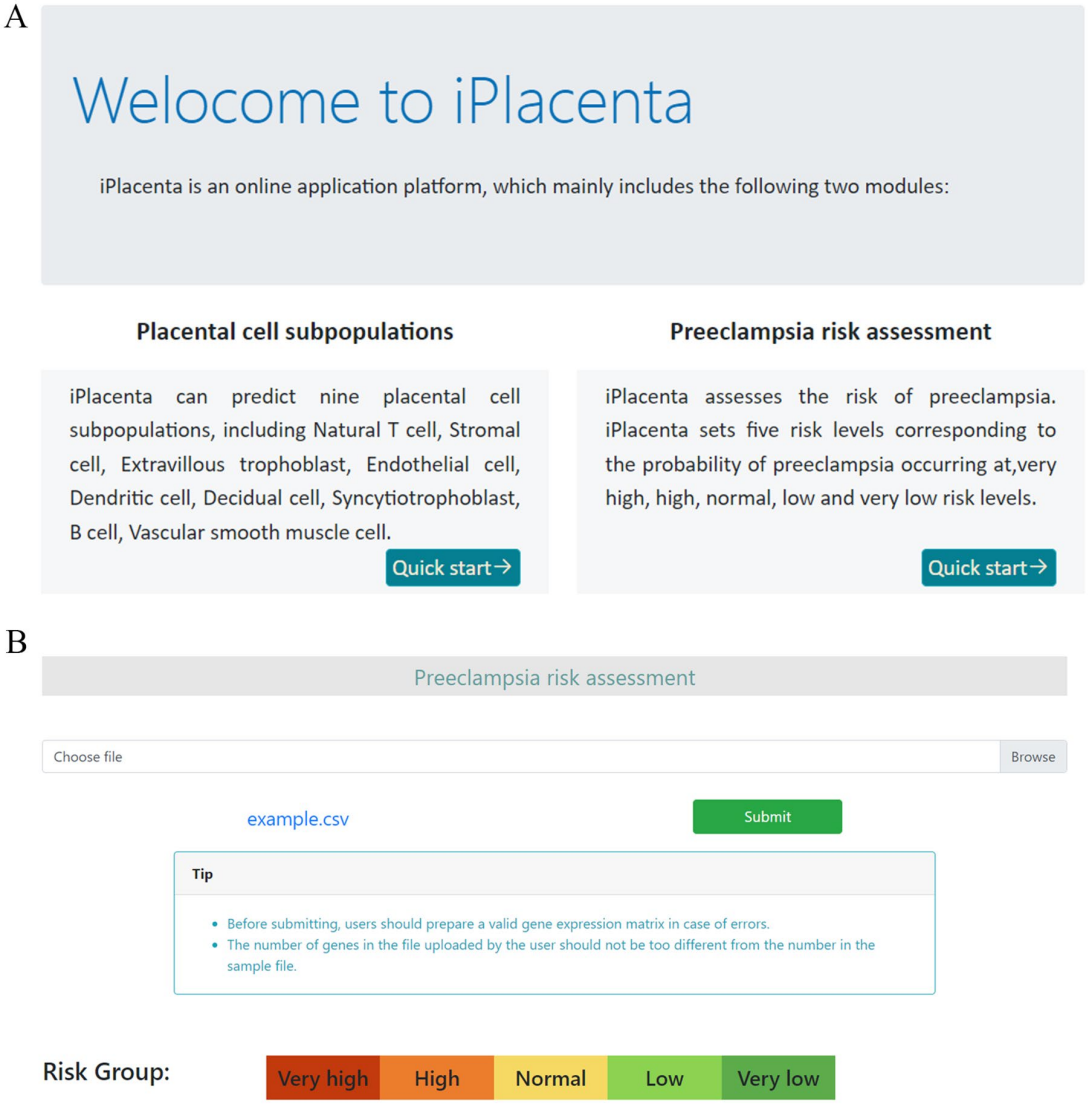
**A** Placental cell subgroups and preeclampsia risk assessment and prediction webserver. **B** Preeclampsia risk prediction module

Step 1. Click the web address http://bioinfor.imu.edu.cn/placenta and the user will see a brief introduction about iPlacenta (Fig. 5A).

Step 2. Click the “Quick Start” button to enter the service module selected by the user. Click the “example” button to download the example data in CSV format. Click the “browse” button, and users can enter the file to be predicted (Fig. 5B).

Step 3. Finally, click the “submit” button to obtain the predicted result.

## Discussion

Placental dysplasia can manifest as miscarriage and complications in late pregnancy, including preeclampsia, fetal growth restriction and intrauterine placental abruption, which are critical for a successful pregnancy and the health of both the fetus and mother [40]. While the cause of PE remains controversial, clinical and pathological studies suggest that the pathogenesis of PE is originated from the placenta [41]. Understanding the placental cell heterogeneity will be helpful for designing more robust and effective cell research and treatment methods. In this study, based on machine learning, different feature selection methods were used to extract the feature information for each cell type of healthy pregnant placenta, annotate specific cell populations and discover significant genes in specific cell populations. We obtained 110 genes that preserved the main patterns of the original biology and achieved satisfactory accuracy. Also known as, these genes faithfully recapitulate cell heterogeneity in placental.

Cellular abnormalities in the placenta of PE affect cell renewal, and the origin of the abnormal cells can be uncovered by comparing the expression levels of specific genes in placental cell subpopulations of PE patients with those of healthy pregnant controls [7]. Along this line, based on the detection of the PE dataset by LASSO, our approach identified 497 genes with the diagnostic capability to distinguish early-onset PE from a normal pregnancy. Notably, some of the 110 placental cell subpopulation marker genes mentioned above were also included in 497 genes, indicating abnormalities in the placental cell subpopulation of PE. In addition, the biological analysis revealed that *C1QB* and *C1QC*, which showed different expression patterns and played a role in the complement and coagulation cascades, might contribute to early-onset PE. Using the learned informative genes, we further developed a predictor to stratify the early-onset PE risk populations and achieved efficient and accurate performance.

However, this study is certainly not without its limitations. Firstly, one major limitation of this study is the small sample size and the absence of external datasets to validate the model, other than cross-validation and independent test sets. The collaborative effort in data collection may facilitate improving the model. Secondly, we identified only 17 genes strongly associated with PE due to the sample size limitations. Going forward, the use of larger datasets or multi-modal features would facilitate the mining more genes related to PE. Despite this potential limitation of the current study, our approach identifies gene features that are important for the identification of PE.

In summary, we demonstrated that single-cell transcriptome-based preeclampsia risk assessment using an ensemble machine learning framework is a valuable asset for clinical decision-making. Our approach is suitable for large-scale preeclampsia screening, realizing early risk warning and screening, which is of great significance for the early control and life intervention of preeclampsia. By predicting risk indicators, medical staff can triage patients, treat them timely and arrange patient treatment plans accordingly, effectively allocate medical resources and reduce mortality. In the aggregate, our study provided a better understanding of the association between PE and abnormal placental cell subpopulations and improved the ability to assess the risk of PE disease.

## Methods

### Dataset construction and preprocessing

Single-cell transcriptome data from healthy pregnancy placentas containing 20,518 cells were collected from the European Bioinformatics Institute (EBI: accession no. EGAS00001002449) [7]. Based on the same processing method used by Tang et al. [7] the data were aligned and quantified using the Cell Ranger single-cell software suite (version 1.0), and sequencing reads were aligned to the hg19 human reference genome using STAR [42], resulting in 35,636 genes. According to the literature survey, nine placental cell subpopulations that have received more attention from biologists were selected for our study. Then, 7178 single-cell transcriptome samples were used to classify nine placental cell subpopulations (Additional file 1: Tables S3 and S5). These single-cell transcriptome samples were randomly divided into a 4809-sample training set and a 2369-sample testing set with a ratio of 7:3.

Single-cell transcriptome data from the placentas of patients with early-onset PE was also collected from EBI (Accession no. EGAS00001002449). The PE prediction dataset was constructed by randomly selecting 7970 early-onset PE samples and combining them with 7178 healthy samples. The same strategy was applied to the segmented PE dataset with 9852 samples in the training set (Healthy 4705 and early-onset PE 5147) and 5305 samples (Healthy 2473 and early-onset PE 2832) in the independent test set (Additional file 1: Tables S4 and S5).

### Model construction of placental cell subpopulations

The placental cell subpopulation gene expression profile was used as input features to train the machine learning model. In exploratory data analysis, important relationships and weights between features could be used to filter out weaker or less relevant information.

The weights of each feature in the training model were evaluated and ranked using MIC [43], ANOVA [44], TURF [45], PCA [46], and F-score [47, 48], respectively. Features with weight scores less than or equal to zero were removed. The IFS [49] was applied to train XGBoost, SVM, KNN, and RFC base models and compare their prediction performance comprehensively.

### Biological analysis and visualization

In addition, the superiority of 110 genes selected by TURF_XGB in predicting cell subpopulations was further analyzed and evaluated. The “TreeExplainer” function was used as an optimized decision tree to calculate the average absolute SHAP value of all features in the model. The integration analysis software implemented in Seurat (version 4.0.3) was used to determine specific cellular subpopulations of marker genes, with all parameters selected by default. Dimensionality reduction was performed by PCA, and visualized by UMAP and tSNE. To identify marker genes in cell clusters, we used the “RidgePlot” function implemented in Seurat to compare cells from a specific cluster with cells in all other clusters. In addition, Scanpy version 1.7.2 was used for PAGA. The python package umap-learn version 0.3.9 was used for UMAP visualization. Specifically, cell trajectory analysis was performed using PAGA implemented on Scanpy for both the original feature dataset and the dataset with only 110 genes, with default parameters.

### Model construction of preeclampsia

LASSO adds the penalty term L1 norm for feature coefficients into the loss function, forcing the coefficients corresponding to these weak features to become zero to achieve sparse solutions [50]. Here, the features with zero coefficients were considered redundant and were discarded, resulting in 497 features selected by LASSO. Ensemble methods are machine learning algorithms that use multiple classifiers and determine the predicted outcome by voting on their predictions. The ensemble methods in MLxtend cover the majority of voting, stacking, and stacked generalization. Based on 497 gene features, “StackingClassifier” was used to ensemble four classifiers, including MLP, SVM, XGBoost, and RFC (The weights assigned to each model is 1). For the training results, we fitted a logistic regression to output predicted probability values. MLxtend is available at https://github.com/rasbt/mlxtend.

### Interpretability of features

SHAP is a method for interpreting the importance of features in machine learning models. In this study, the SHAP algorithm was used to interpret the contribution of each feature in the XGBoost model, and to indicate which features were more likely to be true biomarkers in our ensemble model.

### Risk score card

Based on the logistic regression probability values fitted by the ensemble model, the KS curve was used to depict the overall score. The larger the KS value, the higher the discriminative power of the corresponding threshold in the model. In this study, based on the sample size, equal frequency bins were assigned into five intervals, which corresponded to very high, high, normal, low, and very low risk levels (Additional file 1: Table S2).

### Performance evaluation

Four classic metrics, including accuracy, recall, precision, and F1 measure, were used to quantify the performance of the model, which are defined as follows [51–53]:

1 $$\text{Accuracy}=\frac{TP+TN}{TP+TN+FP+FN}$$

2 $$\text{Recall}=\frac{TP}{TP+FN}$$

3 $$\text{Precision}=\frac{TP}{TP+FP}$$

4 $$\text{F}1\, \text{measure}=\frac{2*\left(precision*recall\right)}{precision+recall}$$

where $TP$, $TN$, $FP$, and $FN$represent the numbers of true positives, true negatives, false positives, and false negatives, respectively.

## Supplementary Information

**Additional file 1: Fig. S1.** Analyze the workflow of the framework. **Fig. S2.** The ridge plots show the new marker genes captured by TURF in each cell subpopulation. **Fig. S3.** Comparison of marker genes selected by TURF using split violin plots. The expression level of marker genes in specific cells is shown on the left, and the total expression level of marker genes in the remaining 8 cell types is shown on the right. **Fig. S4.** Go and KEGG analysis of TURF optimal gene set (A, B, C and D represent biological process (BP), molecular function (MF), cellular component (CC), Kyoto Encyclopedia of Genes and Genomes (KEGG) respectively). **Fig. S5.** Go and KEGG analysis of LASSO optimal feature set (A, B, C and D represent biological process (BP), molecular function (MF), cellular component (CC), Kyoto Encyclopedia of Genes and Genomes (KEGG) respectively. **Table S1.** Performance of five feature selection methods for identifying placental cell subpopulations on four machine learning algorithms (Train dataset). **Table S2.** Preeclampsia risk score card. **Table S3.** Placental cell subpopulation data composition. **Table S4.** Preeclampsia predictor data composition. **Table S5.** Sample information on preeclampsia placenta and control pregnancies. PE was defined as blood pressure ≥ 140/90 mmHg on at least two occasions 4 h apart developing after 20-week gestation with proteinuria of ≥ 300 mg in 24 h, ≥ 30 mg/mmol in protein/creatinine ratio, or two readings of ≥ 2+ on dipstick analysis of midstream or catheter urine specimens if no 24-h collection was available. Only patients not in active labor with delivery by Cesarean section were recruited to avoid cellular contamination from the birth canal and to ensure placental cellular viability.

## Data Availability

All data generated or analyzed during this study are included in this published article.

## Abbreviations

PE: Preeclampsia
scRNA-seq: Single-cell transcriptome
cfRNA: Cell-free RNA
TURF: Tuning ReliefF
XGBoost: eXtreme Gradient Boosting
MIC: Maximal Information Coefficient
PCA: Principal Component Analysis
ANOVA: Analysis of Variance
IFS: Incremental Feature Selection
SVM: Support Vector Machine
RFC: Random Forest Classifier
KNN: K-Nearest Neighbor
TURF_XGB: TURF combined with XGBoost
UMAP: Uniform Manifold Approximation and Projection
SHAP: Shapley’s addition operation
PAGA: Partition-based graphical abstraction
KEGG: Kyoto Encyclopedia of Genes and Genomes
MLP: Multilayer perceptron

## References

[1] Turco MY, Moffett A (2019). Development of the human placenta. Development.

[2] Io S, Kondoh E, Chigusa Y, Kawasaki K, Mandai M, Yamada AS (2020). New era of trophoblast research: integrating morphological and molecular approaches. Hum Reprod Update.

[3] Staff AC (2019). The two-stage placental model of preeclampsia: an update. J Reprod Immunol.

[4] Rana S, Lemoine E, Granger JP, Karumanchi SA (2019). Preeclampsia: pathophysiology, challenges, and perspectives. Circ Res.

[5] Phipps EA, Thadhani R, Benzing T, Karumanchi SA (2019). Pre-eclampsia: pathogenesis, novel diagnostics and therapies. Nat Rev Nephrol.

[6] Staff AC, Benton SJ, von Dadelszen P, Roberts JM, Taylor RN, Powers RW, Charnock-Jones DS, Redman CW (2013). Redefining preeclampsia using placenta-derived biomarkers. Hypertension.

[7] Tsang JCH, Vong JSL, Ji L, Poon LCY, Jiang P, Lui KO, Ni YB, To KF, Cheng YKY, Chiu RWK (2017). Integrative single-cell and cell-free plasma RNA transcriptomics elucidates placental cellular dynamics. Proc Natl Acad Sci U S A.

[8] Vento-Tormo R, Efremova M, Botting RA, Turco MY, Vento-Termo M, Meyer KB, Park JE, Stephenson E, Polanski K, Goncalves A (2018). Single-cell reconstruction of the early maternal-fetal interface in humans. Nature.

[9] Shook LL, Edlow AG (2022). A blood test to predict complications of pregnancy. Nature.

[10] Liu Y, Fan X, Wang R, Lu X, Dang YL, Wang H, Lin HY, Zhu C, Ge H, Cross JC (2018). Single-cell RNA-seq reveals the diversity of trophoblast subtypes and patterns of differentiation in the human placenta. Cell Res.

[11] Moufarrej MN, Vorperian SK, Wong RJ, Campos AA, Quaintance CC, Sit RV, Tan M, Detweiler AM, Mekonen H, Neff NF (2022). Early prediction of preeclampsia in pregnancy with cell-free RNA. Nature.

[12] Rasmussen M, Reddy M, Nolan R, Camunas-Soler J, Khodursky A, Scheller NM, Cantonwine DE, Engelbrechtsen L, Mi JD, Dutta A (2022). RNA profiles reveal signatures of future health and disease in pregnancy. Nature.

[13] Ngo TTM, Moufarrej MN, Rasmussen MLH, Camunas-Soler J, Pan WY, Okamoto J, Neff NF, Liu KL, Wong RJ, Downes K (2018). Noninvasive blood tests for fetal development predict gestational age and preterm delivery. Science.

[14] Zuo Y, Li Y, Chen Y, Li G, Yan Z, Yang L (2017). PseKRAAC: a flexible web server for generating pseudo K-tuple reduced amino acids composition. Bioinformatics.

[15] Shaker B, Kha Mong T, Jung C, Na D (2021). Introduction of advanced methods for structure-based drug discovery. Curr Bioinform.

[16] Mei X, Lee HC, Diao KY, Huang M, Lin B, Liu C, Xie Z, Ma Y, Robson PM, Chung M (2020). Artificial intelligence-enabled rapid diagnosis of patients with COVID-19. Nat Med.

[17] Zhou X, Liu KY, Wong ST (2004). Cancer classification and prediction using logistic regression with bayesian gene selection. J Biomed Inform.

[18] Suresh V, Liu L, Adjeroh D, Zhou X (2015). RPI-Pred: predicting ncRNA-protein interaction using sequence and structural information. Nucleic Acids Res.

[19] Zhu L, Duan G, Yan C, Wang J (2021). Prediction of microbe-drug associations based on chemical structures and the KATZ measure. Curr Bioinform.

[20] Ao C, Yu L, Zou Q (2021). Prediction of bio-sequence modifications and the associations with diseases. Brief Funct Genomics.

[21] Greener JG, Kandathil SM, Moffat L, Jones DT (2022). A guide to machine learning for biologists. Nat Rev Mol Cell Biol.

[22] Ayyad SM, Saleh AI, Labib LM (2019). Gene expression cancer classification using modified K-Nearest neighbors technique. BioSystems.

[23] Jo T (2008). Inverted Index based Modified Version of KNN for text categorization. J Inf Process Syst.

[24] Sainburg T, McInnes L, Gentner TQ (2021). Parametric UMAP embeddings for representation and semisupervised learning. Neural Comput..

[25] Peiffer I, Belhomme D, Barbet R, Haydont V, Zhou YP, Fortunel NO, Li M, Hatzfeld A, Fabiani JN, Hatzfeld JA (2007). Simultaneous differentiation of endothelial and trophoblastic cells derived from human embryonic stem cells. Stem Cells Dev.

[26] Gueguen C, Bouley J, Moussu H, Luce S, Duchateau M, Chamot-Rooke J, Pallardy M, Lombardi V, Nony E, Baron-Bodo V (2016). Changes in markers associated with dendritic cells driving the differentiation of either TH2 cells or regulatory T cells correlate with clinical benefit during allergen immunotherapy. J Allergy Clin Immunol.

[27] Wolf FA, Hamey FK, Plass M, Solana J, Dahlin JS, Gottgens B, Rajewsky N, Simon L, Theis FJ (2019). PAGA: graph abstraction reconciles clustering with trajectory inference through a topology preserving map of single cells. Genome Biol.

[28] Bergen V, Lange M, Peidli S, Wolf FA, Theis FJ (2020). Generalizing RNA velocity to transient cell states through dynamical modeling. Nat Biotechnol.

[29] Theate I, van Baren N, Pilotte L, Moulin P, Larrieu P, Renauld JC, Herve C, Gutierrez-Roelens I, Marbaix E, Sempoux C (2015). Extensive profiling of the expression of the indoleamine 2,3-dioxygenase 1 protein in normal and tumoral human tissues. Cancer Immunol Res.

[30] Garvey SM, Sinden DS, Schoppee Bortz PD, Wamhoff BR (2010). Cyclosporine up-regulates Kruppel-like factor-4 (KLF4) in vascular smooth muscle cells and drives phenotypic modulation in vivo. J Pharmacol Exp Ther.

[31] Broz F, Nehaniv CL, Belpaeme T, Bisio A, Dautenhahn K, Fadiga L, Ferrauto T, Fischer K, Forster F, Gigliotta O (2014). The ITALK project: a developmental robotics approach to the study of individual, social, and linguistic learning. Top Cogn Sci.

[32] Sitras V, Paulssen RH, Gronaas H, Leirvik J, Hanssen TA, Vartun A, Acharya G (2009). Differential placental gene expression in severe preeclampsia. Placenta.

[33] Youssef L, Miranda J, Blasco M, Paules C, Crovetto F, Palomo M, Torramade-Moix S, Garcia-Caldero H, Tura-Ceide O, Dantas AP (2021). Complement and coagulation cascades activation is the main pathophysiological pathway in early-onset severe preeclampsia revealed by maternal proteomics. Sci Rep.

[34] Jia R, Li J, Rui C, Ji H, Ding H, Lu Y, De W, Sun L (2015). Comparative proteomic profile of the human umbilical cord blood exosomes between normal and preeclampsia pregnancies with high-resolution mass spectrometry. Cell Physiol Biochem.

[35] Lokki AI, Heikkinen-Eloranta J (2021). Pregnancy induced TMA in severe preeclampsia results from complement-mediated thromboinflammation. Hum Immunol.

[36] Wang X, Yip KC, He A, Tang J, Liu S, Yan R, Zhang Q, Li R (2022). Plasma olink proteomics identifies CCL20 as a novel predictive and diagnostic inflammatory marker for preeclampsia. J Proteome Res..

[37] Dong Y-M, Bi J-H, He Q-E, Song K (2020). ESDA: an improved approach to accurately identify human snoRNAs for precision cancer therapy. Curr Bioinform.

[38] Liu S, Tang H, Liu H, Wang J (2021). Multi-label learning for the diagnosis of cancer and identification of novel biomarkers with high-throughput omics. Curr Bioinform.

[39] Ao C, Zou Q, Yu L (2021). RFhy-m2G: identification of RNA N2-methylguanosine modification sites based on random forest and hybrid features. Methods..

[40] Suryawanshi H, Morozov P, Straus A, Sahasrabudhe N, Max KEA, Garzia A, Kustagi M, Tuschl T, Williams Z (2018). A single-cell survey of the human first-trimester placenta and decidua. Sci Adv.

[41] Nair TM (2018). Statistical and artificial neural network-based analysis to understand complexity and heterogeneity in preeclampsia. Comput Biol Chem.

[42] Dobin A, Davis CA, Schlesinger F, Drenkow J, Zaleski C, Jha S, Batut P, Chaisson M, Gingeras TR (2013). STAR: ultrafast universal RNA-seq aligner. Bioinformatics.

[43] Albanese D, Filosi M, Visintainer R, Riccadonna S, Jurman G, Furlanello C (2013). Minerva and minepy: a C engine for the MINE suite and its R, Python and MATLAB wrappers. Bioinformatics.

[44] Tukey JW (1949). Dyadic anova, an analysis of variance for vectors. Hum Biol.

[45] Moore JH, White BC (2007). Tuning reliefF for genome-wide genetic analysis.

[46] Alim A, Rafay A, Naseem I (2021). PoGB-pred: prediction of antifreeze proteins sequences using amino acid composition with feature selection followed by a sequential-based Ensemble Approach. Curr Bioinform.

[47] He S, Guo F, Zou Q, Ding H (2020). MRMD2.0: a Python Tool for Machine Learning with feature ranking and reduction. Curr Bioinform.

[48] Liang P, Zheng L, Long C, Yang W, Yang L, Zuo Y (2021). HelPredictor models single-cell transcriptome to predict human embryo lineage allocation. Brief Bioinform.

[49] Ben-Naim A. Elements of information theory: a farewell to entropy: statistical thermodynamics based on information; 2014.

[50] Zhang H, Zhang Q (2021). Potentiality of risk SNPs identification based on GSP theory. Curr Bioinform.

[51] Joshi P, Masilamani V, Ramesh R (2021). An ensembled SVM based approach for predicting adverse drug reactions. Curr Bioinform.

[52] Geete K, Pandey M (2020). Robust transcription factor binding site prediction using deep neural networks. Curr Bioinform.

[53] Ao C, Zhou W, Gao L, Dong B, Yu L (2020). Prediction of antioxidant proteins using hybrid feature representation method and random forest. Genomics.

