# Supplementary material for: A cost-effective machine learning-based method for preeclampsia risk assessment and driver genes discovery
Source: Cell Biosci. 2023 Feb 28;13:41. doi: 10.1186/s13578-023-00991-y (PMC9972636; doi:10.1186/s13578-023-00991-y)
Supplement: Supplementary file 1 — Additional file 1: Fig. S1. Analyze the workflow of the framework. Fig. S2. The ridge plots show the new marker genes captured by TURF in each cell subpopulation. Fig. S3. Comparison of marker genes selected by TURF using split violin plots. The expression level of marker genes in specific cells is shown on the left, and the total expression level of marker genes in the remaining 8 cell types is shown on the right. Fig. S4. Go and KEGG analysis of TURF optimal gene set (A, B, C and D represent biological process (BP), molecular function (MF), cellular component (CC), Kyoto Encyclopedia of Genes and Genomes (KEGG) respectively). Fig. S5. Go and KEGG analysis of LASSO optimal feature set (A, B, C and D represent biological process (BP), molecular function (MF), cellular component (CC), Kyoto Encyclopedia of Genes and Genomes (KEGG) respectively. Table S1. Performance of five feature selection methods for identifying placental cell subpopulations on four machine learning algorithms (Train dataset). Table S2. Preeclampsia risk score card. Table S3. Placental cell subpopulation data composition. Table S4. Preeclampsia predictor data composition. Table S5. Sample information on preeclampsia placenta and control pregnancies. PE was defined as blood pressure ≥ 140/90 mmHg on at least two occasions 4 h apart developing after 20-week gestation with proteinuria of ≥ 300 mg in 24 h, ≥ 30 mg/mmol in protein/creatinine ratio, or two readings of ≥ 2+ on dipstick analysis of midstream or catheter urine specimens if no 24-h collection was available. Only patients not in active labor with delivery by Cesarean section were recruited to avoid cellular contamination from the birth canal and to ensure placental cellular viability. [file 13578_2023_991_MOESM1_ESM.docx]

**A Cost-Effective Machine Learning-based Method for Preeclampsia Risk Assessment and Driver Genes Discovery**

Hao Wang^1,2,^**^†^**, Zhaoyue Zhang^3,^**^†^**, Haicheng Li^1,2,^**^†^**, Jinzhao Li^1^, Hanshuang Li^1^, Mingzhu Liu^1,2^, Pengfei Liang^1^, Qilemuge Xi^1^, Yongqiang Xing^4,*^, Lei Yang^5,*^, Yongchun Zuo^1, 2,*^

^1^The State Key Laboratory of Reproductive Regulation and Breeding of Grassland Livestock, College of Life Sciences, Inner Mongolia University, Hohhot, 010070, China.

^2^Digital College, Inner Mongolia Intelligent Union Big Data Academy, Inner Mongolia Wesure Date Technology Co., Ltd. Hohhot, 010010, China.

^3^School of Life Science and Technology, Center for Informational Biology, University of Electronic Science and Technology of China, Chengdu, 610054, China.

^4^School of Life Science and Technology, Inner Mongolia University of Science and Technology, Baotou, 014010, China.

^5^College of Bioinformatics Science and Technology, Harbin Medical University, Harbin, 150081, China.

**^†^**These authors contributed equally to this work.

* Corresponding authors: Yongchun Zuo, State Key Laboratory of Reproductive Regulation and Breeding of Grassland Livestock, College of Life Sciences, Inner Mongolia University, Hohhot, 010070, China, Email: [yczuo@imu.edu.cn](mailto:yczuo@imu.edu.cn); Lei Yang, College of Bioinformatics Science and Technology, Harbin Medical University, Harbin, 150081, China, Email: [leiyang@hrbmu.edu.cn](mailto:leiyang@hrbmu.edu.cn); Yongqiang Xing, School of Life Science and Technology, Inner Mongolia University of Science and Technology, Baotou, 014010, China, Email: xingyongqiang1984@163.com.


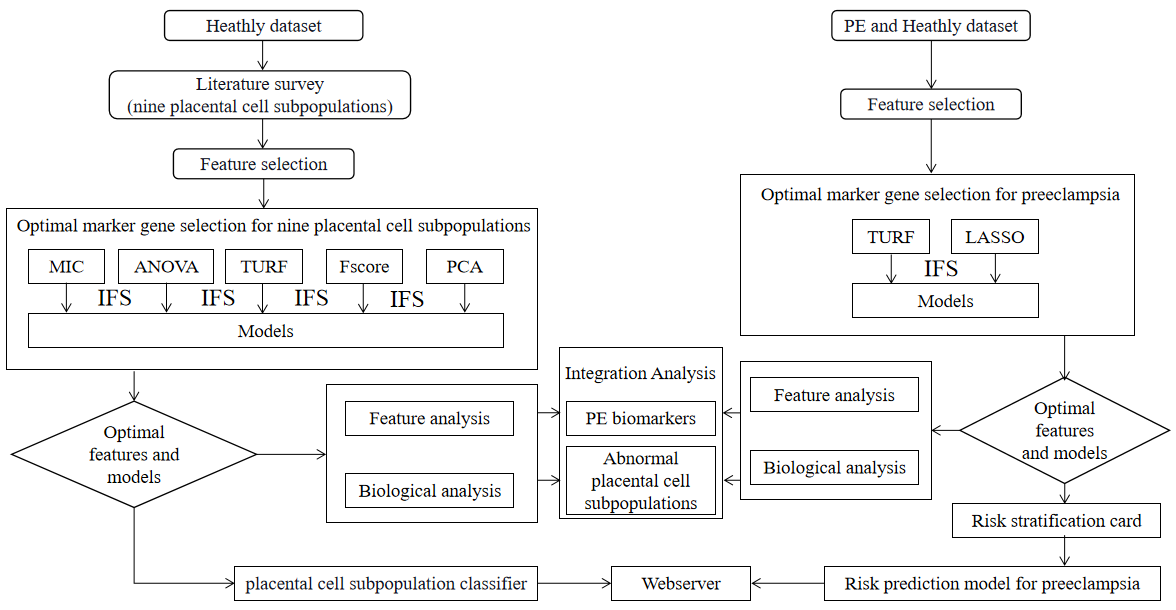


**Fig. S1** Analyze the workflow of the framework.


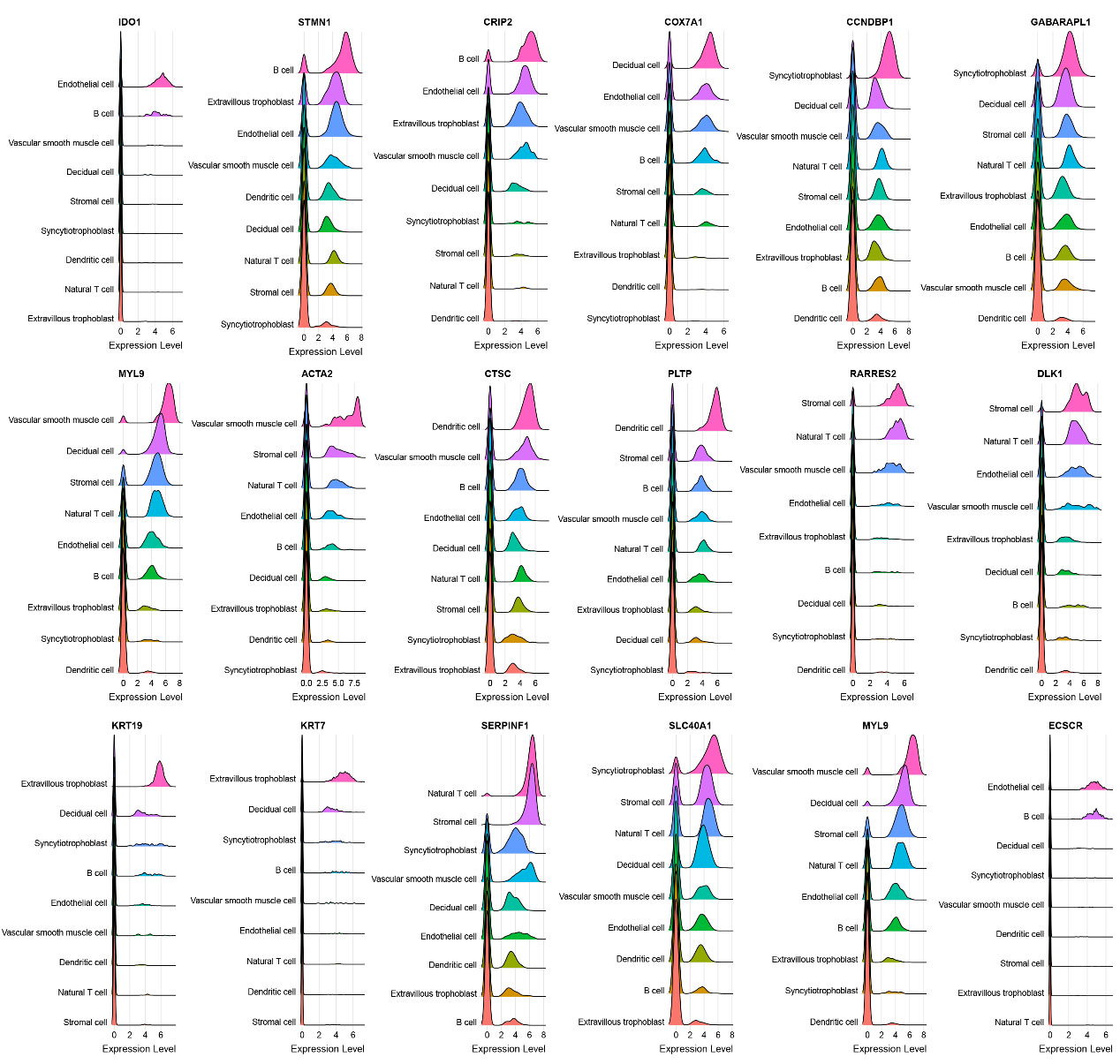


**Fig. S2** The ridge plots show the new marker genes captured by TURF in each cell subpopulation.


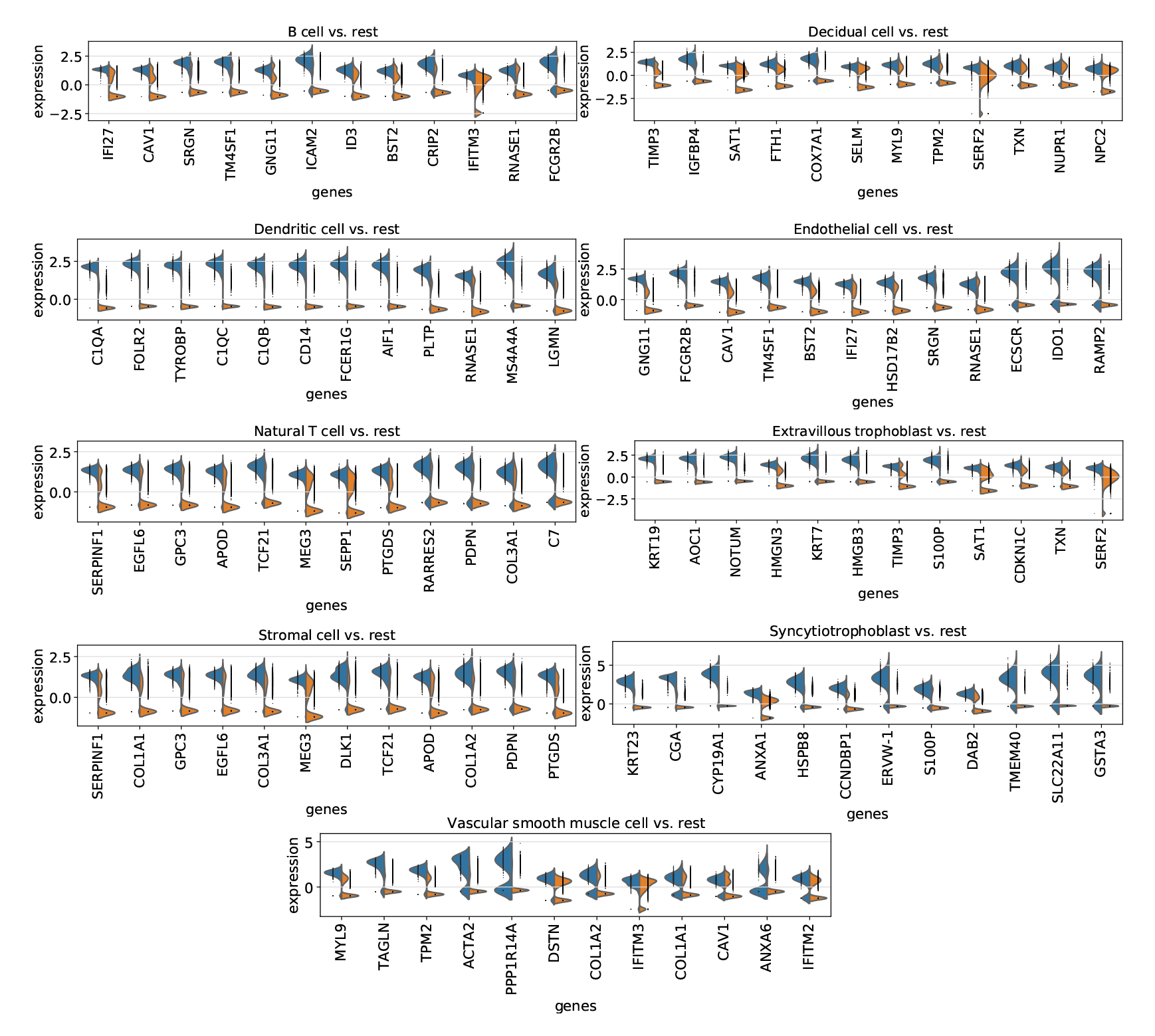


**Fig. S3** Comparison of marker genes selected by TURF using split violin plots. The expression level of marker genes in specific cells is shown on the left, and the total expression level of marker genes in the remaining 8 cell types is shown on the right.


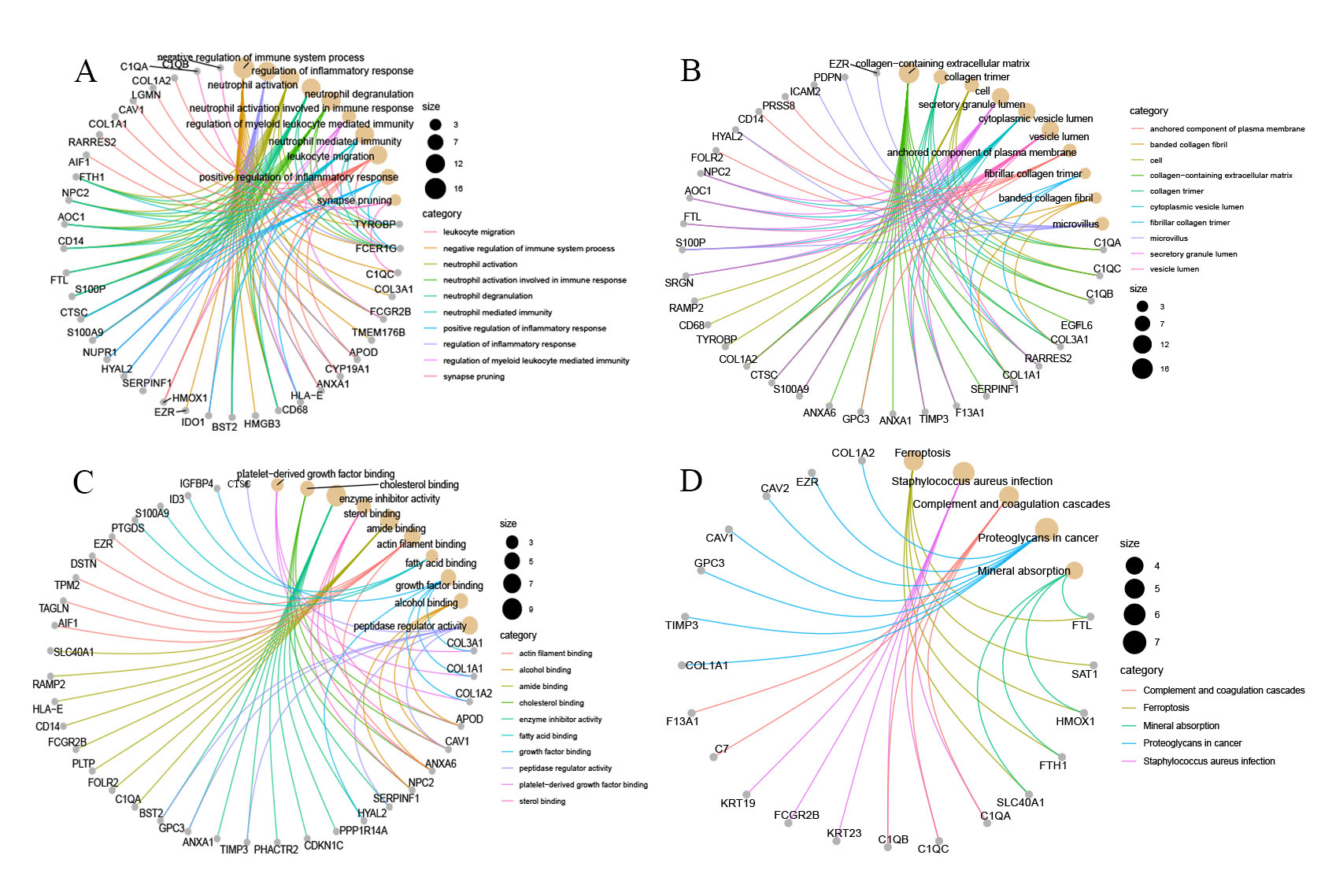


**Fig. S4** Go and KEGG analysis of TURF optimal gene set (A, B, C and D represent biological process (BP), molecular function (MF), cellular component (CC), Kyoto Encyclopedia of Genes and Genomes (KEGG) respectively)


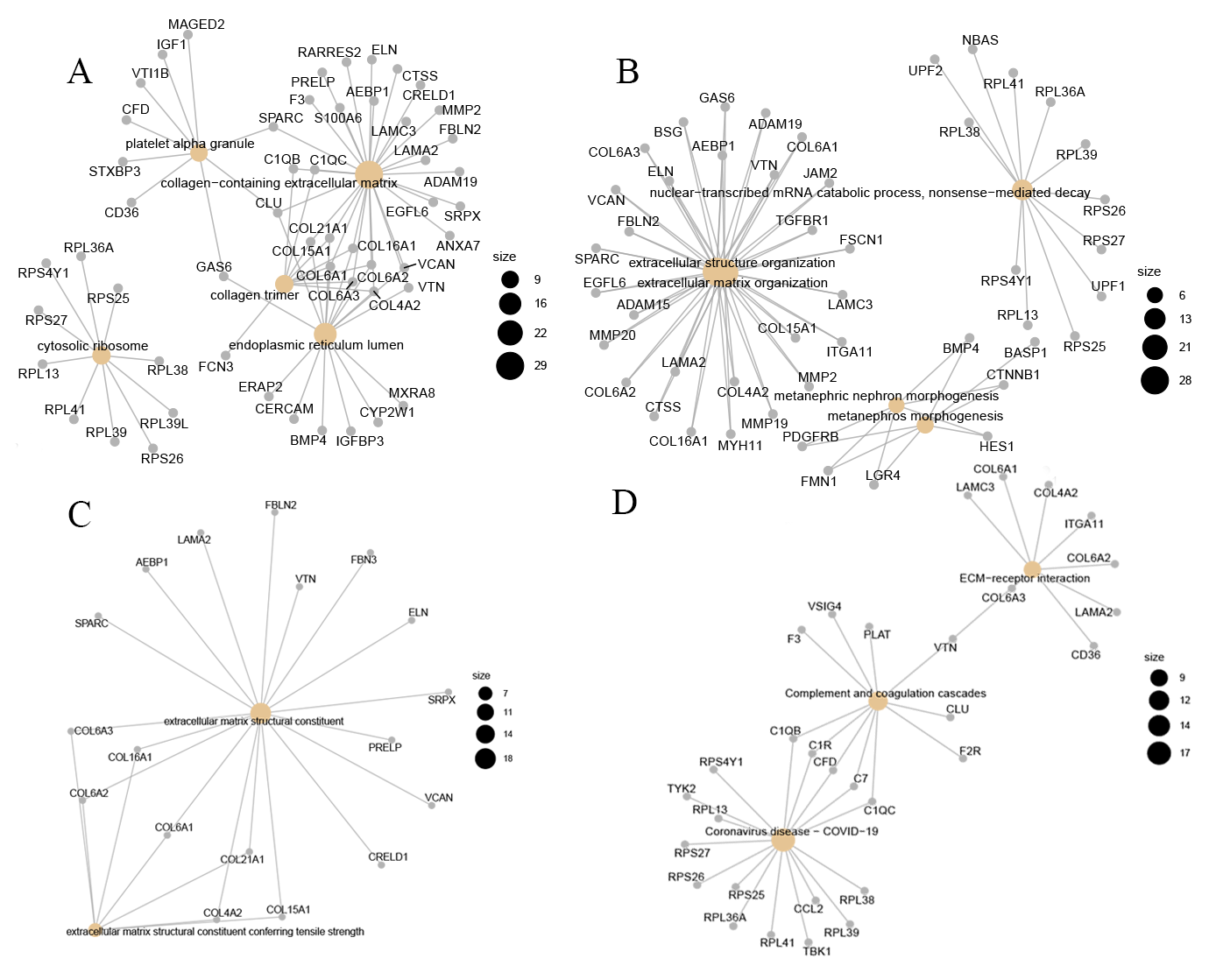


**Fig. S5** Go and KEGG analysis of LASSO optimal feature set (A, B, C and D represent biological process (BP), molecular function (MF), cellular component (CC), Kyoto Encyclopedia of Genes and Genomes (KEGG) respectively.

**Table S1** Performance of five feature selection methods for identifying placental cell subpopulations on four machine learning algorithms (Train dataset).

| Base classifier | Feature selection | Feature numbers | Accuracy |
| --- | --- | --- | --- |
| KNN | PCA | 160 | 73.79% |
| RFC | PCA | 3000 | 91.22% |
| SVM | PCA | 1200 | 89.22% |
| XGBoost | PCA | 2000 | 92.05% |
| KNN | MIC | 210 | 88.83% |
| RFC | MIC | 260 | 92.41% |
| SVM | MIC | 160 | 92.45% |
| XGBoost | MIC | 310 | 92.02% |
| KNN | TURF | 110 | 88.22% |
| RFC | TURF | 310 | 92.49% |
| SVM | TURF | 210 | 92.61% |
| XGBoost | TURF | 110 | 92.89% |
| KNN | F-score | 310 | 86.31% |
| RFC | F-score | 610 | 92.32% |
| SVM | F-score | 710 | 91.99% |
| XGBoost | F-score | 410 | 92.07% |
| KNN | ANOVA | 360 | 83.65% |
| RFC | ANOVA | 810 | 92.79% |
| SVM | ANOVA | 710 | 92.53% |
| XGBoost | ANOVA | 460 | 91.11% |

**Table S2** Preeclampsia risk score card.

| Start | End | Proportion  of health | Proportion of PE | KS_value | Risk  group |
| --- | --- | --- | --- | --- | --- |
| 0 | 20 | 0.3750 | 0.0000 | 0.3750 | very low |
| 20 | 40 | 0.7471 | 0.0028 | 0.7443 | low |
| 40 | 60 | 0.9932 | 0.1500 | 0.8432 | normal |
| 60 | 80 | 1.0000 | 0.5713 | 0.4286 | high |
| 80 | 100 | 1.0000 | 1.0000 | 0.0000 | very high |

**Table** **S3** Placental cell subpopulation data composition.

| Cell cluster | Training set  cell number | Testing set  cell number | Total number |
| --- | --- | --- | --- |
| Natural T cell | 663 | 337 | 1000 |
| Stromal cell | 649 | 351 | 1000 |
| Extravillous trophoblast | 671 | 329 | 1000 |
| Endothelial cell | 686 | 314 | 1000 |
| Dendritic cell | 681 | 319 | 1000 |
| Decidual cell | 660 | 316 | 976 |
| Syncytiotrophoblast | 269 | 149 | 418 |
| B cell | 279 | 121 | 400 |
| Vascular smooth muscle cell | 251 | 133 | 384 |
| Total number | 4809 | 2369 | 7178 |

**Table** **S4** Preeclampsia predictor data composition.

| Cell cluster | Training set cell number | Testing set cell number | Total number |
| --- | --- | --- | --- |
| Healthy | 4705 | 2473 | 7178 |
| preeclampsia | 5147 | 2832 | 7979 |
| Total number | 9852 | 5305 | 15157 |

**Table S5** Sample information on preeclampsia placenta and control pregnancies. PE was defined as blood pressure ≥140/90 mmHg on at least two occasions 4 h apart developing after 20-week gestation with proteinuria of ≥300 mg in 24 h, ≥30 mg/mmol in protein/creatinine ratio, or two readings of ≥2+ on dipstick analysis of midstream or catheter urine specimens if no 24-h collection was available. Only patients not in active labor with delivery by Cesarean section were recruited to avoid cellular contamination from the birth canal and to ensure placental cellular viability.

| **Sample_ID** | **Group** | **Gestation age, week** | **Condition** |
| --- | --- | --- | --- |
| M12604 | PE | 31^+2^ | Early-onset |
| M12630 | PE | 32^+4^ | Early-onset |
| M12632 | PE | 29^+6^ | Early-onset |
| M12612 | PE | 28^+1^ | Early-onset |
| M12548 | Healthy | 38^+2^ | Healthy |
| M12491 | Healthy | 38 | Healthy |
| M12475 | Healthy | 38 | Healthy |
| M12551 | Healthy | 38 | Healthy |
